# Association of peripheral manifestation of brain‐derived neurotrophic factor with depression: A meta‐analysis

**DOI:** 10.1002/brb3.2581

**Published:** 2022-05-05

**Authors:** Sagun Tiwari, Lili Qi, John Wong, Zhenxiang Han

**Affiliations:** ^1^ Department of Neurology and Rehabilitation Seventh People's Hospital of Shanghai University of TCM Shanghai P. R. China; ^2^ International Education College Shanghai University of Traditional Chinese Medicine Shanghai P. R. China; ^3^ Department of Emergency Medicine Yueyang Hospital of Integrated Traditional Chinese and Western Medicine Shanghai University of TCM Shanghai P. R. China; ^4^ School of Nursing and Department of Occupational Therapy MGH Institute of Health Professions Boston Massachusetts USA

**Keywords:** biomarker, brain‐derived neurotrophic factor, major depressive disorder, neurotrophic factors, neurotrophin

## Abstract

**Background:**

The relationship between brain‐derived neurotrophic factor (BDNF) and depression is a hot topic in research as several results of preclinical and clinical studies have shown controversial results. Our meta‐analysis aims to evaluate and update the current status of peripheral BDNF with depression.

**Methods:**

We performed a meta‐analysis by comprehensively searching PubMed and Web of Science for English‐language literature from inception to 1st of June 2020. The search terms included brain‐derived neurotrophic factor or BDNF in combination with depression, without year restriction. Using STATA software, data were pooled using a random‐effects model.

**Results:**

In our literature search, 24 studies involving 1130 depressed patients and 1378 healthy individuals met our inclusion criteria. The results of our meta‐analysis showed that the peripheral levels of BDNF levels significantly decreased in depression than nondepressed healthy controls (SMD = −0.89, 95% CI = −1.41, −0.38, *p* < .0001); however, the significant heterogeneity among studies (Q = 740.91, *I* ^2 ^= 96.8; *p *< .001) was discovered. Trim‐and‐fill estimations for the adjustment of publication bias indicated that publication bias had no impact on our results. Our sub‐group analysis showed that a history of depression and alcohol consumption had an effect on the level of BDNF. In addition, age and gender did not affect the heterogeneity of BDNF in the meta‐analysis.

**Conclusions:**

Although decreased peripheral expression of BDNF certainly presents a risk of depression, we cannot find a definite relationship between the peripheral level of BDNF with depression to use BDNF as a reliable biomarker to assess the depression in clinical practice. We propose that future research should consider all the factors affecting BDNF and assess the level of proBDNF and mBDNF separately while evaluating the patients with depression objectively.

## Graphical abstract

A meta‐analysis study of the peripheral manifestation of brain‐derived neurotrophic factor in relation to depression.

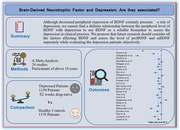

## 1 INTRODUCTION

In recent years, depression has received much attention due to its high prevalence, recurrence rate, and considerable impact on the economy. Depression is a profound debilitating disease that is projected to be the leading cause of the burden of disease worldwide by 2030, resulting in a high socioeconomic burden to society due to severe limitations on psychosocial functioning and diminished quality of life (Liu et al., 2020; Malhi & Mann, 2018). Multiple risks and causative factors of depression give rise to the following symptoms: depressed mode, anhedonia, irritable behavior, neuro‐vegetative symptoms, suicidal thoughts, and other organic and cognitive complications (Fried et al., 2016; H. S. Kim & Moore, 2019). Despite advancements in the field, the underlying pathophysiology and mechanism of depression are still inconclusive compared to other diseases. Moreover, proper and reliable assessment methods such as the measurement of biomarkers need to be introduced so that physicians can objectively determine a patient's status in depression.

Presently, there are no concrete biomarkers to assess depression clinically. However, many scientists have proposed that brain‐derived neurotrophic factor (BDNF) might play a significant role in the pathophysiological mechanism of depression. BDNF has a notable involvement in neurogenesis, neuroplasticity, cognitive functions, and other vital functions of the brain that have been contributed to depression (Colucci‐D'Amato et al., 2020; Failla et al., 2016; Kowiański et al., 2018; Yang et al., 2020). According to the neurotrophin hypothesis, altered neurogenesis, which may regulate memory and emotion, has been proposed to be the result of the lowered level of BDNF (Schinder & Poo, 2000). Many previous animal, postmortem, and clinical studies have suggested that BDNF level decreases in patients with depression‐like behavior and impaired neurogenesis phenomena (Emon et al., 2020; Lorenzetti et al., 2020; Murínová et al., 2017; Nunes et al., 2018; Sheldrick et al., 2017). Additional studies have been conducted to establish the relationship between BDNF and depression, and some of the results have demonstrated that BDNF might act as a potential biomarker for depression, as a low level of BDNF was found in depressed patients (Polyakova, Stuke, et al., 2015; Rana et al., 2020). However, other studies have presented not only inconsistent results but also contradictory results to the aforementioned claim (Castrén & Kojima, 2017; Miao et al., 2020; Miranda et al., 2019; Zhou et al., 2017).

Because of the inconsistent results in the literature, this article aims to address the controversial issue regarding the BDNF with depression. Based on the hypothesis of “peripheral as a window to the brain,” our study examined peripheral BDNF level as a proxy for its central nervous system (CNS) expression and compared the peripheral level of BDNF between depressed patients and healthy individuals (Gejl et al., 2019; Molendijk et al., 2014). We conducted this meta‐analysis to expand our current knowledge in this issue by performing a comprehensive analysis of all the currently available data related to BDNF and depression. In addition, we aim to determine whether BDNF could act as a biomarker for depression in light of any controversial inconsistent results between the relationship of peripheral BDNF and depression.

## 2 METHODS

**FIGURE 1:**
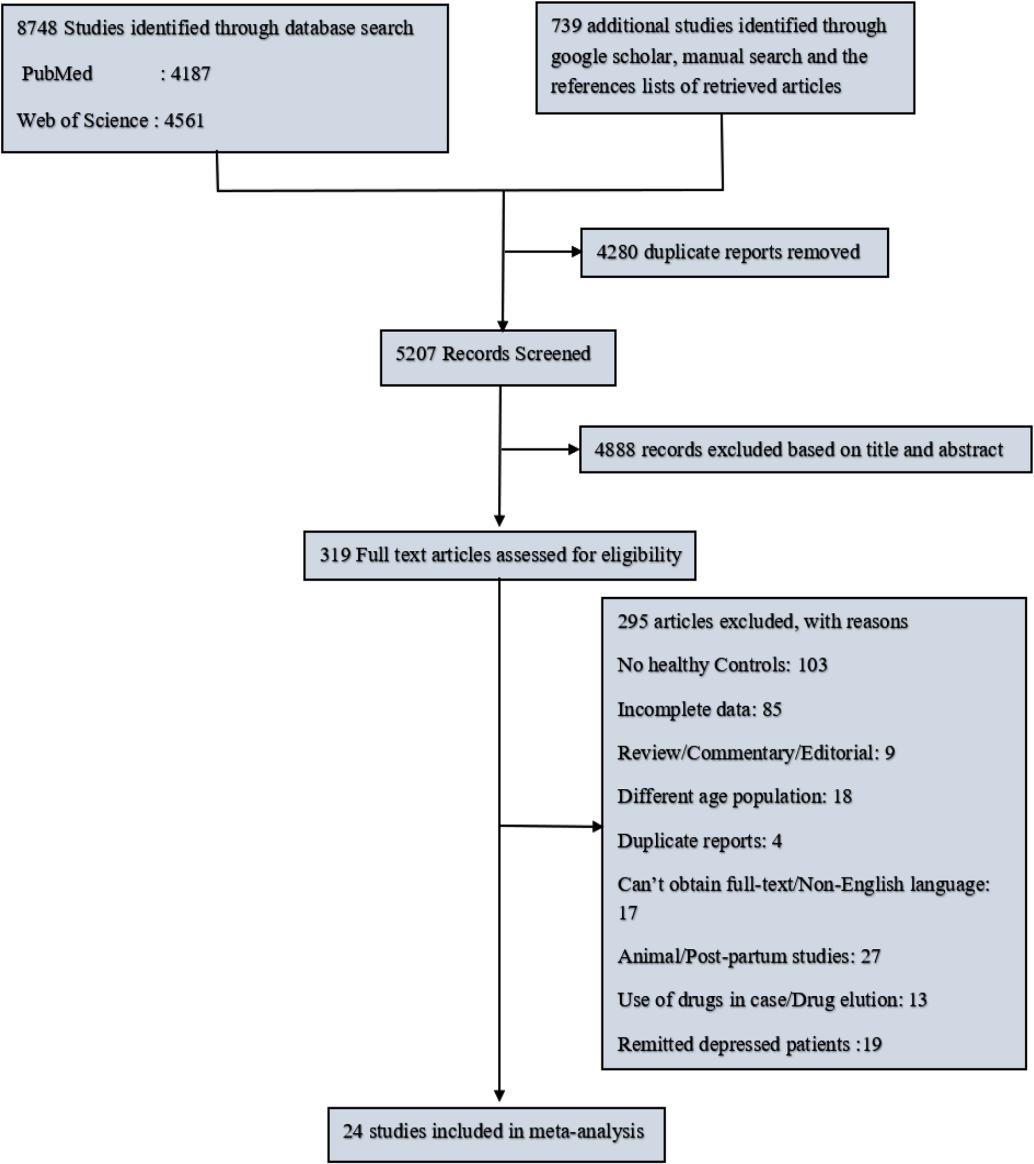
Preferred Reporting Items for Systematic Reviews and Meta‐Analyses (PRISMA) flow diagram of the literature search

Our study followed the Preferred Reporting Items for Systematic Reviews and Meta‐Analyses (PRISMA) reporting guidelines. The literature search, data extraction, and inclusion decisions were conducted by two authors (Sagun Tiwari and Zhenxiang Han) (Figure 1).

### 2.1 Data sources, searches, and eligibility criteria

We performed a comprehensive systematic search of English‐language publications using PubMed and Web of Science, which have been published until the 1st of June 2020, to identify research articles on BDNF concentration in depressed patients without any treatment and healthy controls. Additional searches on Google Scholar, manual search, and the reference lists of relevant articles were also performed. The search terms used for the database search included brain‐derived neurotrophic factor or BDNF in combination with depression. Original peer‐reviewed empirical human studies (> 18years) that reported data on peripheral blood levels of BDNF in patients with depression undergoing at least 2 weeks drug‐naïve or drug‐free treatment and healthy controls were included, independent of the methodological characteristics of the sample or study. Excluded from our analysis were articles with secondary analyses of the same data and those with a sample size below 10.

### 2.2 Study selection and data extraction

After the titles and abstracts of the search results were screened, the full‐text articles were obtained to further assess eligibility. Potential errors and doubts were resolved by discussion among all researchers. Sample sizes, mean (SD) BDNF concentrations (depressed and healthy), publication year, names of the first authors, sample source (plasma/serum), assay type, mean age, and gender distribution were extracted from the selected studies (Table 1). In the case of missing, incomplete, and ambiguous data, the corresponding authors of included studies were contacted for clarification.

**TABLE 1:** Description of the studies included in the meta‐analysis

Study /year	Sample (total)	Samples (DP)	Samples (HC)	Gender (%total male)	Gender (% male) (DP)	Gender (% male) (HC)	Age (total mean)	Mean age (DP)	Mean age (HC)	BDNF mean concentrations (DP)	BDNF mean concentrations (HC)	BDNF SD Concentrations (DP)	BDNF SD Concentrations (HC)	Unit	Sample source	Assay
(Emon et al., 2020)	167	85	82	41.27	43.52	39.02	32.25	33.37 ± 1.10	31.13 ± 1.82	578.62	723.77	44.65	78.36	Pg/ml	Serum	ELISA
(Aldoghachi et al., 2019)	412	206	206	31.55	31.55	31.55	N/A	N/A	N/A	5168	7287	339.9	342.1	Pg/ml	Plasma	ELISA
(Hsieh et al., 2019)	110	48	62	N/A	N/A	N/A	N/A	N/A	N/A	5.6	7.9	4.5	3.2	Ng/ml	Serum	ELISA
(Druzhkova et al., 2019)	76	33	43	43	42	44	31.7	32.89 ± 7.82	30.51 ± 5.5	26.93	26.58	8.12	6.91	Ng/ml	Serum	ELISA
(Halappa et al., 2018)	64	13	51	N/A	N/A	N/A	33.72	34.60 ± 8.85	32.84 ± 9.14	19.32	23.58	6.22	6.81	Ng/ml	Serum	N/A
(Jiang et al., 2017)	70	35	35	42.85	31.42	54.28	50.35	43.97 ± 13.33	56.74 ± 4.59	17380	23420.16	5161.24	8525.71	Pg/ml	Serum	ELISA
(Chiou & Huang, 2017)	142	71	71	21.12	21.12	21.12	35.35	37.4 ± 10.5	33.3 ± 5.4	10	13.3	7	7.8	Ng/ml	Serum	ELISA
(Hui et al., 2016)	66	32	34	0	0	0	30.48	31.21 ± 3.21	29.75 ± 2.99	1.46	1.84	0.2	0.22	Ng/ml	Serum	ELISA
(Polyakova, Sander et al., 2015)	103	21	82	47.76	33.33	62.19	70.6	71.2 ± 4.5	70 ± 4.1	25.8	25.2	5.4	5.9	μ/L	Serum	ELISA
(de Azevedo Cardoso et al., 2014)	240	120	120	20.83	20.83	20.83	23.77	23.74 ± 3.33	23.81 ± 3.27	4.65	7.37	4.18	2.31	Ng/ml	Serum	ELISA
(Fornaro et al., 2015)	62	30	32	22.5	20	25	46.75	48.27 ± 9.6740	45.23 ± 11.623	8.107	9.705	1.934	1.585	Ng/ml	Serum	ELISA
(Lee et al., 2014)	69	34	35	31.84	29.41	34.28	43.9	47 ± 18.4	40.8 ± 9.4	21.2	24.89	7.21	7.38	Ng/ml	Serum	ELISA
(Harvey et al., 2013) (male)	101	41	60	100	100	100	42.97	41.87 ± 7.89	44.07 ± 8.01	1301	1170.75	750.7	519.51	Pg/ml	Serum	ELISA
(Harvey et al., 2013) (female)	99	48	51	0	0	0	45.39	45.44 ± 8.03	45.35 ± 7.76	1480.26	1698.44	631.4	603.84	Pg/ml	Serum	ELISA
(Kotan et al., 2012)	80	40	40	20	20	20	34.5	35 ± 8	34 ± 8	1577	1624	5105	329	Pg/ml	Serum	ELISA
(Chu et al., 2012)	134	12	122	100	100	100	82.1	82.4 ± 4.4	81.8 ± 5	115.1	548.8	57.2	370.6	Pg/ml	Plasma	ELISA
(Oral et al., 2012)	79	39	40	31.6	28.2	35	26.75	26.3 ± 4	27.2 ± 4	1.75	1.91	0.35	0.36	Ng/ml	Serum	ELISA
(Papakostas et al., 2013)	79	36	43	48.21	63.88	32.55	36.25	42.5 ± 9.8	30 ± 8.6	15174	10096	8163	6946	Pg/ml	Serum	ELISA
(Hung et al., 2010)	108	55	53	36.13	34.54	37.73	N/A	N/A	N/A	5.24	5.5	3.7	3.58	Ng/ml	Serum	ELISA
(Eker et al., 2010)	47	25	22	24	28	20	30.9	32.1 ± 9.3	29.7 ± 6.4	21.7	27	6.6	5.7	Ng/ml	Serum	ELISA
(Matrisciano et al., 2009)	42	21	21	48.69	52.38	45	37.1	42.4 ± 8	31.8 ± 5.9	35.4	64.1	15.2	13.1	Ng/ml	Serum	ELISA
(Piccinni et al., 2008)	30	15	15	16.66	13.33	20	41.95	47 ± 10.8	36.9 ± 9.2	2900	5400	1900	2300	Pg/ml	Plasma	ELISA
(Y.‐K. Kim et al., 2007)	62	32	30	41.97	40.62	43.33	44.25	47.47 ± 14.7	41.03 ± 7.81	875.8	889.4	663.02	611.3	Pg/ml	Plasma	ELISA
(Aydemir et al., 2005)	20	10	10	20	20	20	35.8	31.8 ± 14.3	39.8 ± 7.1	17.9	31.6	9.1	8.6	Ng/ml	Serum	ELISA
(Gonul et al., 2005)	46	28	18	29.16	25	33.33	35.6	35.5 ± 8.1	35.7 ± 5.8	20.8	26.8	6.7	9.3	Ng/ml	Serum	ELISA

Abbreviations: BDNF, brain‐derived neurotrophic factor; DP, depression patients; HC, healthy controls.

### 2.3 Statistical analysis

Statistical analyses were performed using the STATA software (version 16.0; Stata Corporation, College Station, TX, USA). We examined the relationship between peripheral BDNF and depression based on standardized mean differences (SMDs) and 95% confidence intervals (CIs) reported in each study. Statistical heterogeneity between studies was evaluated by the Q statistic and *I* ^2^ statistic, and substantial heterogeneity was determined when *p* < .1. In the presence of statistical heterogeneity, a random effect model was used for the analysis. In the absence of statistically significant heterogeneity, the SMDs of the fixed effect model are calculated. Publication bias was examined by visual inspection of a funnel plot with additional trim and fill estimation. Sensitivity analysis was performed to test the stability of the results by excluding each study iteratively. Meta‐regression and subgroup analysis were performed. All reported *p*‐values are two‐sided, and *p*‐values ≤0.05 were considered statistically significant for all the included studies.

## 3 RESULTS

First, we performed a systematic search in which we yielded a total number of 9487 studies. Then, after removing 4280 duplicate studies, we screened 5207 studies for a preliminary screening phase based on the titles and abstracts to further exclude 4888 studies because they were irrelevant to our study. The remaining 319 studies were retrieved for a more detailed full‐text analysis in which we excluded 295 studies based on our eligibility criteria. Finally, 24 studies were included in our meta‐analysis study, which involves 1130 patients with depression and 1378 healthy controls (Table 1). In addition, we contacted several corresponding authors to complement incomplete data from their studies.

**FIGURE 2:**
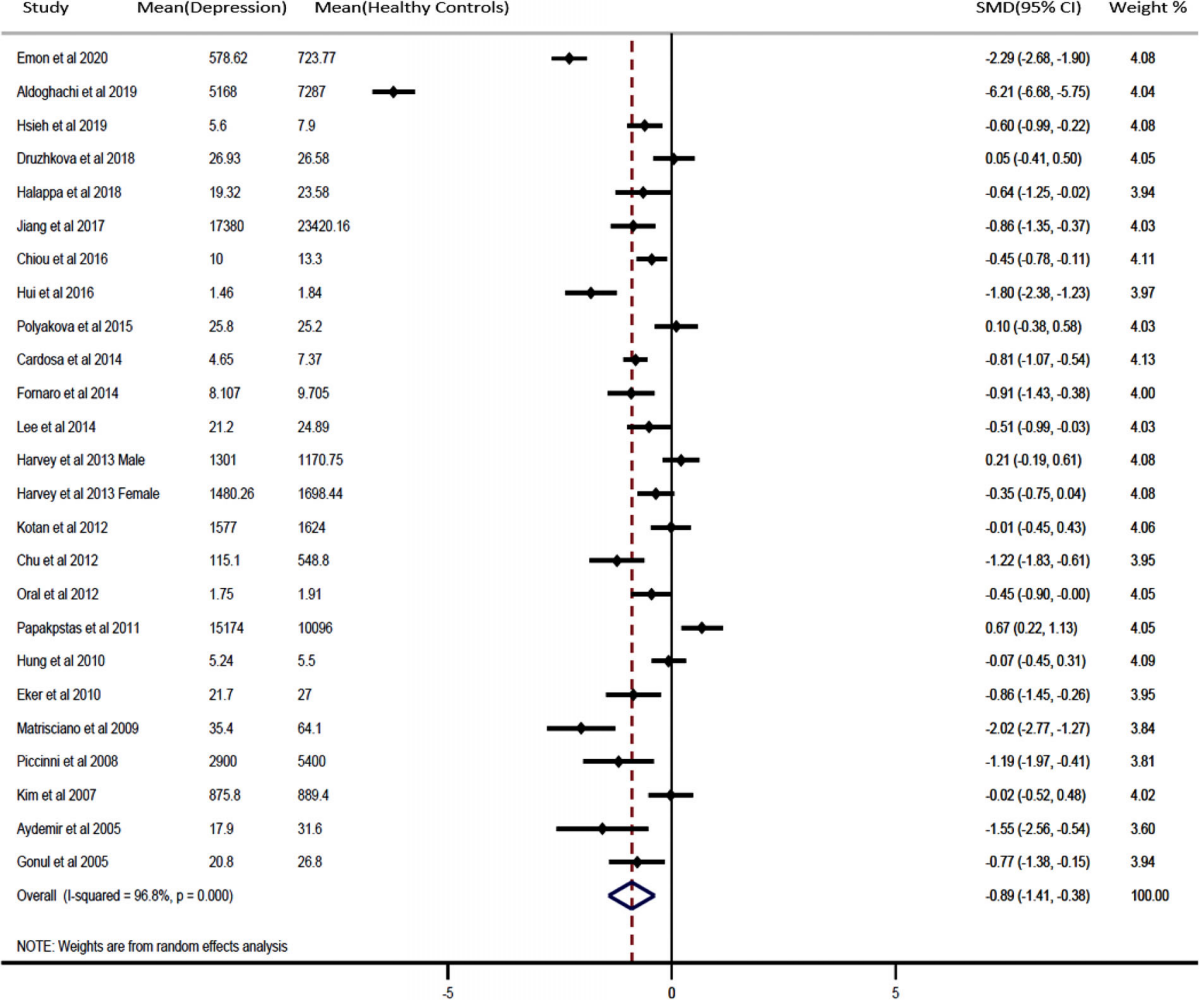
Forest plot for random‐effects meta‐analysis differences in peripheral levels of brain‐derived neurotrophic factors are shown between the patients with depression and healthy controls. The sizes of the squares are proportional to study weights. Diamond marker indicates pooled effect size

A random‐effects model meta‐analysis was performed on the extracted 24 studies representing 1130 patients with depression without any treatment and 1378 healthy individuals (controls). As shown in Figure 2, our findings demonstrated that in patients with depression without any treatment, the peripheral levels of BDNF levels were significantly decreased relative to levels in nondepressed healthy controls (SMD = −0.89, 95% CI = −1.41, −0.38, *p* < .0001). According to sensitivity analysis, no single study had a substantial impact on the notable difference in blood BDNF levels between depression patients and healthy controls (Figure S1). In our meta‐analysis, however, we discovered significant heterogeneity among studies (*Q* = 740.91, *I* ^2 ^= 96.8; *p *< .001).

### 3.1 Subgroup analysis and meta‐regression

**FIGURE 3:**
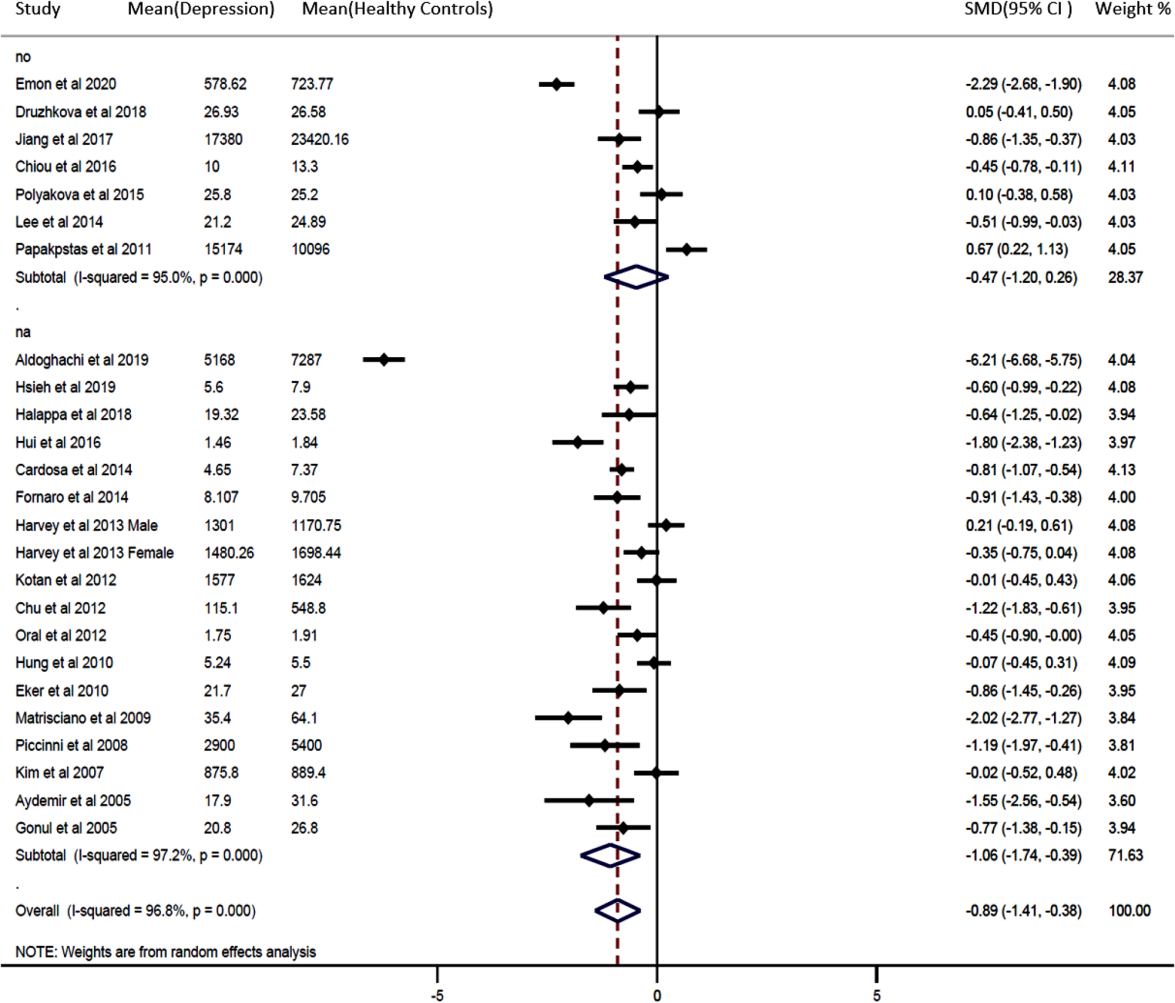
Forest plot for sub‐group analysis (history of depression). Pooled results compare peripheral brain‐derived neurotrophic factor (BDNF) between the patients with depression and healthy controls. The study design is stratified into the history of depression and not available of history of depression. The sizes of the squares are proportional to study weights. Diamond marker indicates pooled effect sizes

**FIGURE 4:**
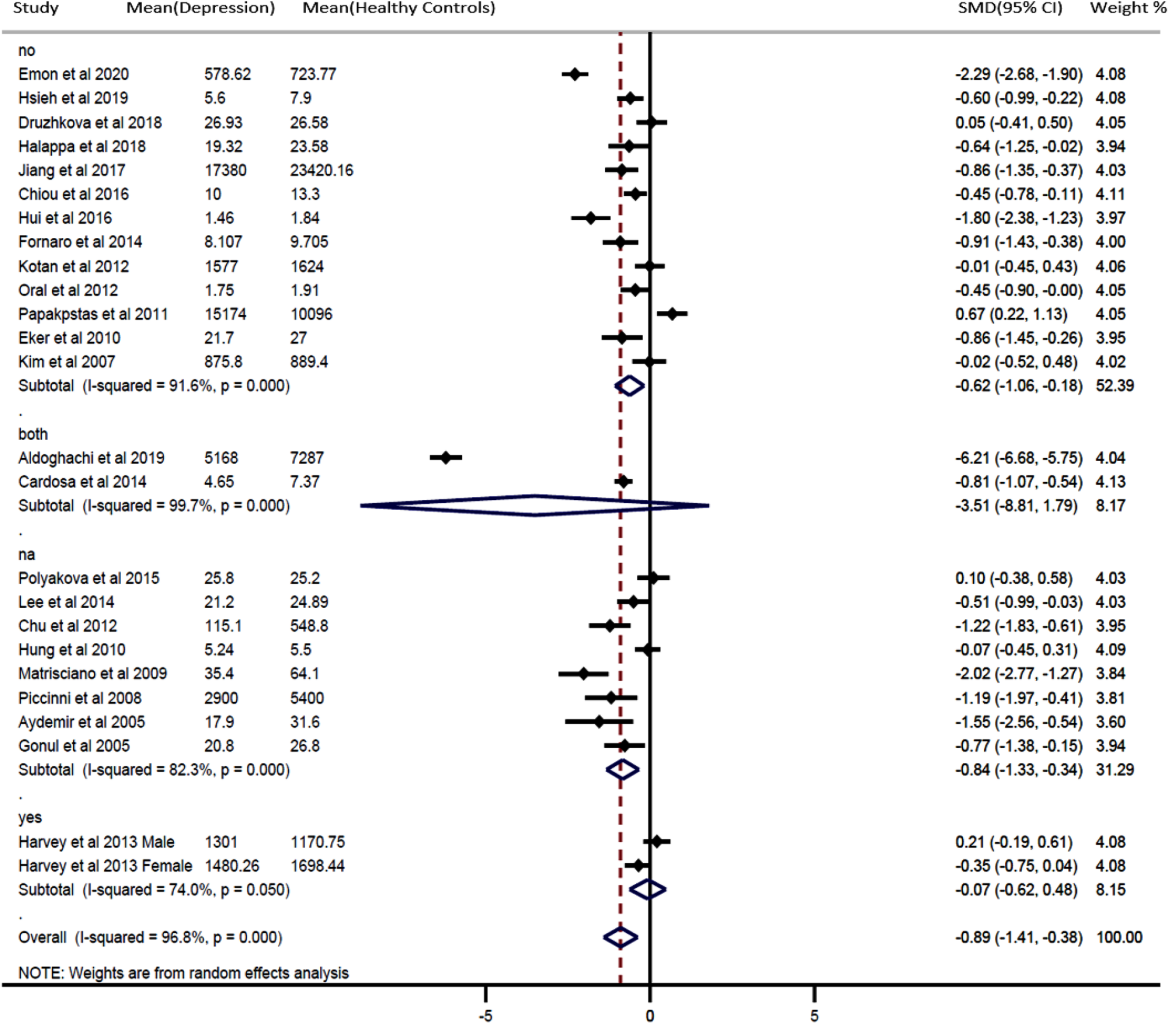
Forest plot for subgroup analysis (alcohol consumption). Pooled results compare peripheral brain‐derived neurotrophic factor (BDNF) between the patients with depression and healthy controls. The study design is stratified into alcohol consumption, not available, and no alcohol consumption. The sizes of the squares are proportional to study weights. Diamond marker indicates pooled effect sizes

We performed subgroup analyses on alcohol consumption and history of depression (Figures 3 and 4). Depressed patients with no alcohol consumption had significantly decreased blood BDNF levels compared with healthy controls (SMD = −0.62, 95% CI = −1.06, −0.18; *p* = .000). In addition, patients with a history of depression had significantly decreased blood BDNF levels compared with healthy controls (SMD = −2.24, 95% CI = −4.90, −0.42; *p* = .000). Furthermore, the results of meta‐regression analyses demonstrated that continuous variables such as patient age and gender had no effect on the significant heterogeneity between studies (Figures S2 and S 3). The publication was evaluated through the visual inspection of the funnel plot, which was found to be mildly asymmetric. Hence, the trim and fill method for adjusting the publication bias was used to evaluate the number of missing studies, resulting in six missing studies. After including those hypothetical six missing studies, the results remained unchanged, and the differences between groups were statistically significant (−1.100 [95% CI: −1.182, −1.019]; *p* = .000) under the fixed‐effects model; (−1.182 [95% CI: −1.655, −0.709]; *p* = .000) under random‐effects model.

## 4 DISCUSSION

Prior studies related to BDNF and depression have produced conflicting results that fail to show conclusively whether BDNF is directly or indirectly proportional to depression (Castrén & Kojima, 2017; Druzhkova et al., 2019; Miao et al., 2020; Miranda et al., 2019; Papakostas et al., 2013; Zhou et al., 2017). As a result, in this current study, we explored the possible reason behind the conflicting outcome across studies of BDNF and depression by evaluating and/or comparing the BDNF concentrations in both drug‐naïve or drug‐free patients and healthy controls. We were able to confirm that the peripheral expression of BDNF concentrations is lower in drug‐naïve or drug‐free depressed patients than in healthy individuals, which have been previously reported in individual studies that have been previously published (Chiou & Huang, 2017; de Azevedo Cardoso et al., 2014; Fornaro et al., 2015; Kishi et al., 2017; Molendijk et al., 2014; Zhou et al., 2017).

Although our study validates altered expression in the depressed patient, we also discovered a significant amount of heterogeneity between studies, significantly influencing the BDNF expression in drug‐naïve or drug‐free depressed patients. We speculate that a high heterogeneity might have resulted from between‐sample characteristics, such as alcohol consumption and cigarette smoking, sleep‐related issue, circadian variation, socioeconomic status, and differences in the severity of patient samples’ clinical features such as a history of depression (Bus et al., 2012; Miranda et al., 2019; Wessels et al., 2020). These heterogeneous variables are interdependent in a complex manner, and any disruption of this complex mechanism may give rise to depression (Athira et al., 2020; Beirão et al., 2020; McEwen & Akil, 2020). It was impossible to differentiate all these variables in our study. The 24 different studies in our analysis included subjects with unique physiological or pathological stressors affecting each of them in a different way, and all subjects were included as a whole despite these individual differences, thereby affecting the results of our study. While not all those variables were available from all the selected studies, we were able to perform subgroup analyses on alcohol consumption and history of depression. According to our results, drug‐naïve or drug‐free depressed patients with alcohol have lower BDNF concentrations than those without alcohol. Specifically, a history of depression in drug‐naïve or drug‐free depressed patients was negatively correlated with blood BDNF levels. Altogether, alcohol consumption and history of depression had a significant effect on our results. Meta‐regression analyses, however, of other variables such as age and gender did not affect the level of BDNF (Figures S2 and S 3), even though many of the previously published studies have reported that BDNF concentrations considerably decrease with increasing age and in females compared to males (Albert, 2015; Geldsetzer et al., 2019; Kreinin et al., 2015; Weisbrod et al., 2019; Zis et al., 2017).

In our analysis, trim‐and‐fill estimations were used to assess the impact of publication bias (Figure S4). If trim‐and‐fill estimation reveals numerous missing reports, suggesting publication bias, it would be unreliable to accept research that states that peripheral levels of BDNF are decreased in depression. Our study revealed that six studies were missing, which when imputed the result remain unchanged, suggesting that publication bias has little effect on our result (−1.100 [95% CI: −1.182, −1.019]; *p* = .000) under the fixed‐effects model; (−1.182 [95% CI: −1.655, −0.709]; *p* = .000) under random‐effects model.

Our results led to the conclusion that BDNF cannot be used as reliable clinical biomarker for depression. However, in atrophy, synaptic disconnection, and irregular functioning of depression circuits, BDNF has been shown to play an important role (Arosio et al., 2021; Yang et al., 2020). Decreased neurotrophic support through chronic stress negatively impacts the survival of neurons and hampers the functions of the hippocampus, so BDNF may be involved in promoting the progression of depression symptoms (Arosio et al., 2021; Pitsillou et al., 2020). Recent studies have also concluded that BDNF is correlated with depression (Emon et al., 2020; He et al., 2019; Yang et al., 2020). In our previous study, we have also concluded that BDNF is a valuable predictor of poststroke depression but not for poststroke anxiety (Han et al., 2020). Although BDNF may have an important involvement in the pathophysiological mechanism of depression, it does not seem to be a reliable indicator of functional outcome in the general population. Furthermore, no association between BDNF polymorphisms and depression has been found in large‐scale studies (Kishi et al., 2017; Peters et al., 2020; Tsai, 2018; Youssef et al., 2018). For decades, it was believed that monoamine deficiency was the cause of depression's pathophysiology. But, later on, there was no universal efficacy in this theory and then the BDNF hypothesis was proposed, and this hypothesis is now being used in many studies. However, several irregularities and inconsistencies have been discovered in this current theory throughout the research. Thus, we suggest that now may be the time to reassess this theory just like the scientific community did with the monoamine hypothesis of depression.

Inflammation has been proposed as one of the pathological mechanisms in major depression disorder (MDD). Many studies have supported that gene products of BDNF (proBDNF and mBDNF) might have a potential role to play in developing depression (Arteaga‐Henríquez et al., 2019; Pitsillou et al., 2020; Wang et al., 2019). The Yin and Yang hypothesis of BDNF has provided significant insight into depression (Jaggar et al., 2019; Lu et al., 2005). Various studies have reported that the precursor proBDNF and the mature protein mBDNF can elicit opposite biological effects through binding to p75^NTR^ and Trk receptors, respectively (Bothwell, 2019; Gibon et al., 2016; Porcher et al., 2018). Therefore, it may be necessary to differentiate proBDNF and mBDNF and study their action or mechanism in depression state. Recently, one article has presented evidence that mBDNF could be the biomarker to assess depression, through their newly developed ELISA kit. Nevertheless, the mechanism of how mBDNF is decreased in MDD still has not been elucidated (Lin et al., 2021), but a thorough investigation of the action of proBDNF/mBDNF may potentially present a major breakthrough in our understanding of MDD.

**FIGURE 5:**
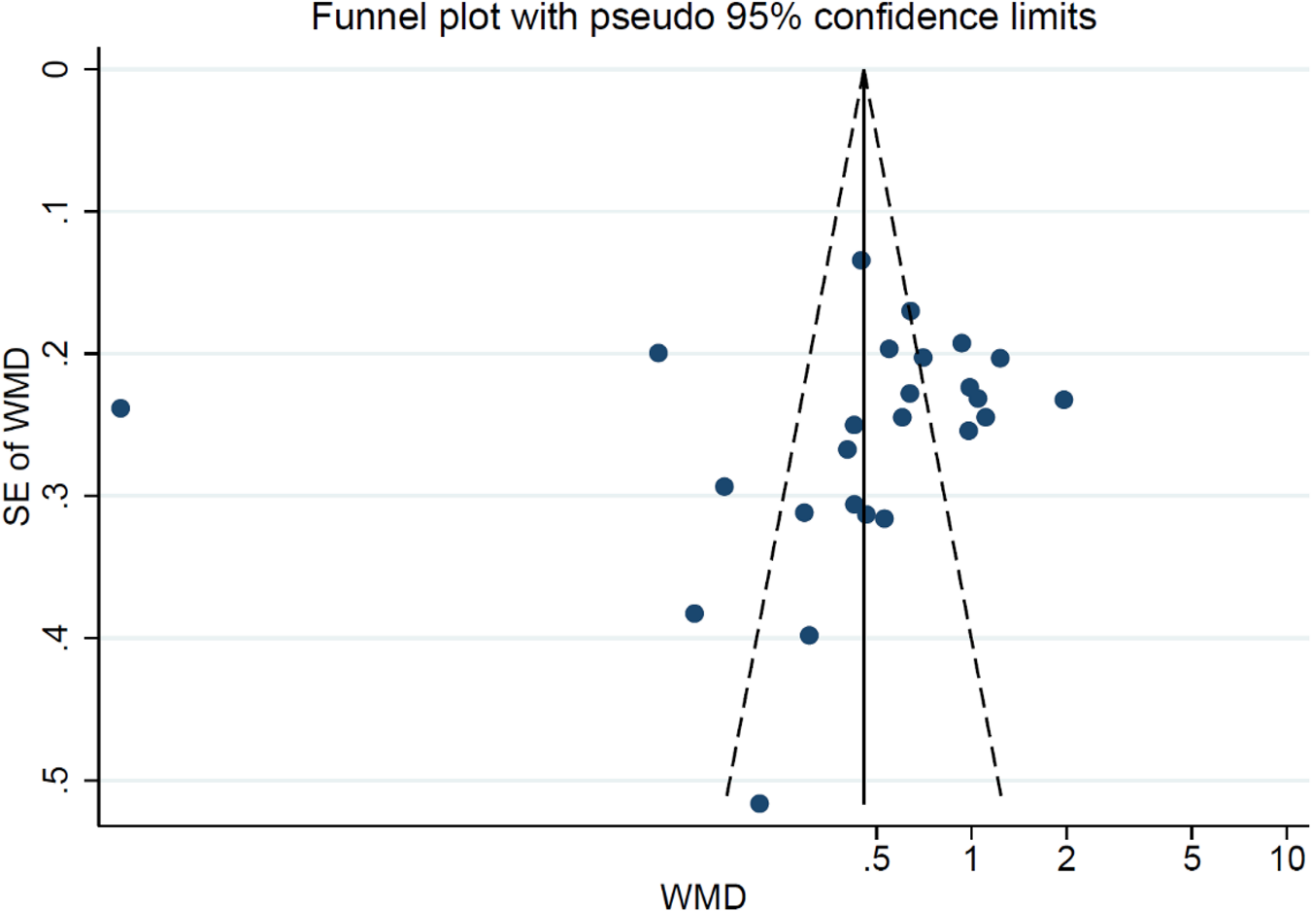
Funnel plot Publication bias in studies comparing peripheral brain‐derived neurotrophic factor (BDNF) levels between patients with depression and healthy controls. The plots describe the effect size (Hedges *g* statistic) of studies against their precision (inverse of SE). Data markers indicate individual studies

The main limitation of our meta‐analysis is that we were unable to examine all the determinants of peripheral BDNF concentrations apart from the history of depression, alcohol consumptions, age, and gender because these variables that might alter BDNF expression were missing from a majority of studies. Second, we did not compare the data between depression patients with drugs and drug naïve depressed patients and compared them to the healthy controls. Third, we only selected English literature and published articles in a peer‐reviewed journal, and it is very possible that there is a higher number of studies published with positive results than ones with negative ones, resulting in a significant publication bias, although we did not find any publication bias in our study (Figure 5). Fourth, BDNF values were not investigated before and after treatment on depressed patients. Fifth, we adopted a combination of depression diagnoses including major, mild and minor, to refer to depression as a whole. Considering that depression itself is a complex heterogeneous disease with many variations in its etiopathogenesis and high heterogeneity, this study proposes that a lowered expression of BDNF blood levels in depression patients may not only be due to the pathophysiological mechanism of depression but also due to other confounding factors, which we should look into detail in future studies.

## 5 CONCLUSION

Decreased peripheral expression of BDNF certainly presents a risk of depression, but in our sub‐group analysis, alcohol consumption and history of depression, unlike age and gender, may have influenced the outcome of our results, a novel finding that has not been previously reported. Because it is difficult to confirm a definite relationship between the peripheral level of BDNF with depression, we advocate that we should rather focus on studying mBDNF and proBDNF separately, not BDNF as a whole, in depression. Altogether, future research should consider all the potential determinants of BDNF (sampling, sociodemographic, lifestyle indicators, and diseases) and evaluate the ratio of proBDNF and mBDNF to generate a full picture of the complex relationships between these factors.

## CONFLICT OF INTEREST

The authors declare no conflict of interest.

## AUTHOR CONTRIBUTIONS

Zhenxiang Han is the principal investigator of this study, overseeing study design, data collection, interpretation, and manuscript preparation. Sagun Tiwari had an important role in study design, data collection, interpretation, and manuscript preparation. Lili Qi contributed to data analysis. John Wong assisted for data interpretation and manuscript preparation.

### PEER REVIEW

The peer review history for this article is available at https://publons.com/publon/10.1002/brb3.2581.

## Data Availability

The original contributions presented in the study are included in the article/supplementary material, further inquiries can be directed to the corresponding author.
